# Evidence of changes to skeletal muscle contractile properties during the initiation of disease in the ageing guinea pig model of osteoarthritis

**DOI:** 10.1186/2046-2395-2-15

**Published:** 2013-12-01

**Authors:** Daniel P Tonge, Ronald G Bardsley, Tim Parr, Rose A Maciewicz, Simon W Jones

**Affiliations:** 1 Nutritional Sciences, School of Biosciences, Sutton Bonington Campus, Sutton Bonington, University of Nottingham, Nottingham LE12 5RD, England; 2 Respiratory & Inflammation, AstraZeneca, Charnwood R&D, Loughborough, Leicestershire LE11 5RH, England; 3 MRC-ARUK Centre for Musculoskeletal Ageing Research, School of Immunity and Infection, Queen Elizabeth Hospital, University of Birmingham, Edgbaston B15 2WB, England

**Keywords:** Ageing, Age-related, Dunkin Hartley, Myosin heavy chain, Osteoarthritis

## Abstract

**Background:**

Osteoarthritis (OA) is the most common joint disorder in the world and represents the leading cause of pain and disability in the elderly population. Advancing age remains the single greatest risk factor for OA. Several studies have characterised disease development in the guinea pig ageing model of OA in terms of its joint histopathology and inflammatory cytokine profile. However, the quadriceps muscle has yet to be studied in relation to age-related disease onset or early disease progression. Therefore, we examined whether the initiation of OA in the Dunkin Hartley guinea pig is associated with changes in the quadriceps skeletal muscle. Male Dunkin Hartley guinea pigs (N = 24) were group housed with free access to standard guinea pig chow and water. At 2, 3, 5 and 7 months of age, six animals were selected based on their proximity to the median weight of the cohort. OA severity was graded at each time point by the assessment of toluidine blue stained step coronal sections of the total knee joint. Serum CTX II was measured as a potential biomarker of OA severity. Myosin Heavy Chain (MHC) isoforms were determined by a validated real-time PCR assay. Oxidative and glycolytic potential was determined in quadriceps homogenates via the measurement of ICDH and LDH activity.

**Results:**

Initiation of OA in the DH strain guinea pig occurred between 2 and 3 months of age and progressed until 7 months when the final analyses were conducted. Serum CTX II significantly decreased during this early period of OA initiation and levels were unrelated to the histopathological severity of knee OA at any of the time points assessed. MHC mRNA measurements revealed a significant elevation in MHC IIX mRNA (associated with fast-twitch skeletal muscle fibres) coincident with the initiation of OA at 3 months of age, with preliminary findings suggestive of a positive correlation to OA severity at this time point.

**Conclusions:**

These preliminary findings suggest that disease initiation in the ageing guinea pig model of OA is not associated with overt quadriceps muscle atrophy but instead is coincident with altered expression of mRNAs associated with quadriceps skeletal muscle contractile properties (specifically fast-twitch MHC IIX).

## Background

Osteoarthritis (OA) is the most common joint disorder in the world and represents the leading cause of pain and disability in the elderly population [1-3]. Advancing age remains the single greatest risk factor for OA in susceptible joints, with the prevalence of knee OA specifically increasing for each decade of life after the age of 60 [4,5]. Advancing age is also associated with functional changes to the skeletal muscle system including decreased mass, strength and proprioception [3-8]. These functional changes result from sarcopenia, a process which includes progressive denervation, atrophy due to disuse, and the accumulation of connection tissue [5,9].

It is known that patients with knee OA exhibit muscle weakness [1,10-17], which is one of the most frequent and earliest reported symptoms [18]. It primarily affects the quadriceps muscle with little or no evidence of hamstring weakness [11], resulting in a reduced quadriceps to hamstring ratio [19]. Quadriceps to hamstring ratio perturbations may be further accentuated in some instances by hypertrophy of the hamstring muscle in addition to quadriceps dysfunction [20]. Historically, muscle weakness has been considered a secondary effect in knee OA, resulting from disuse of the affected joint due to the presence of pain and/or inflammation, and therefore has received little attention with regards to its involvement in the initiation or progression of OA. However, growing evidence suggests that quadriceps weakness may precede the onset of radiographic evidence of OA and pain [13], and be directly involved in its pathogenesis [14]. Firstly, quadriceps weakness is reported in those patients with radiographic signs of knee OA in the absence of pain, suggesting that the muscle weakness is unlikely to be due to disuse of a painful joint [21]. Secondly, quadriceps weakness is noted in a number of patient groups who are susceptible to developing knee OA; for example, patients who have gait abnormalities resulting in increased knee loading [22], patients with anterior cruciate ligament insufficiencies [20] and, most commonly, patients who have undergone partial meniscectomy surgery as a treatment of medial meniscal tears [23].

In attempting to identify and develop new therapeutics for OA, the Dunkin Hartley guinea pig model has been extensively used by ourselves and others since it develops OA spontaneously with advancing age and has several clear parallels with the human condition both during initiation and disease progression [24]. For example, OA initially develops predominantly on the medial aspect of the tibial condyle, with involvement of the medial femoral condyle only in response to disease progression [24-30]. This finding replicates the human situation where approximately 75% of the load is passed through the medial aspect of the knee [25]. The development of OA in the Dunkin Hartley strain has also been strongly associated with increasing age and body mass [31] as with the human condition [1]. Furthermore, similarities between the Dunkin Hartley model and human OA have also been described at the molecular level. For example, the development of human knee OA has been associated with the expression of collagenase 1 and collagenase 3, also known as matrix metalloproteinases 1 and 13 respectively, at the site of OA development [32,33]. Importantly, both collagenase 1 and 3 are highly expressed in the Dunkin Hartley guinea pig model [34].

Several studies have previously characterised the age-related development of OA in the guinea pig in terms of its joint histopathology [28] and inflammatory cytokine profile [35]. However, the quadriceps muscle has yet to be studied in relation to primary disease onset or early disease progression. We hypothesised that the initiation of knee OA would be associated with changes to the quadriceps skeletal muscle group. Further, these changes may manifest as changes in gross muscle mass, subtle changes to the contractile and metabolic potential of this muscle group, or a combination of the two processes. With the aim being to further characterise the Dunkin Hartley guinea pig as a model for age-related human knee OA, we performed a preliminary study using a small cohort of animals to assess changes in the quadriceps muscle group during the initiation and early progression of OA in the guinea pig model. In order to fully characterise the age-related development of OA in this species, it is critical to assess the molecular and pathological changes that occur earliest during disease initiation. Dunkin Hartley guinea pigs have a lifespan of approximately 4 years, reaching sexual maturity from approximately 45 days after birth. With this in mind, four discrete ages were chosen at which we hypothesised the animals would be free from disease (2 months), developing initial pre-osteoarthritic changes (3 months) and progressing to moderate OA during early adulthood (5 and 7 months). At all ages, we characterised contractile and metabolic-associated factors in the quadriceps muscle and determined OA severity through histopathological staining of knee joint sections. Subtle changes in factors associated with muscle contractility were determined using a set of oligonucleotide primers developed and qualified specifically for this purpose [36] (Table 1).

**Table 1:** Oligonucleotide primer sequences for quantitative PCR assessment of Guinea pig myosin heavy chain mRNA

**Gene name**	**Forward primer (5'–3')**	**Reverse primer (5'–3')**	**Amplicon size (bp)**
MyH1 (MHC IIx)	TTCATCCAAATGCAGGAAAG	TCTTTATCTCAAAAGTCATAAATACAA	90
MyH2 (MHC IIa)	TGTGGAATGACCAGAGCAAG	CCTTTGCAATAGGGTAGGACA	85
MyH4 (MHC IIb)	TCCATCTACTGCTGCAACG	ACTCTGCAGATTTTATTTCCTTG	93
MyH7 (MHC I)	AAGTATCGCAAGGCTCAA	CCTTTCCTTAATTCCAAGC	129

See Tonge et al. for a comprehensive assessment of primer specificity [36].

## Results and discussion

### Animal weight parameters

**Figure 1:**
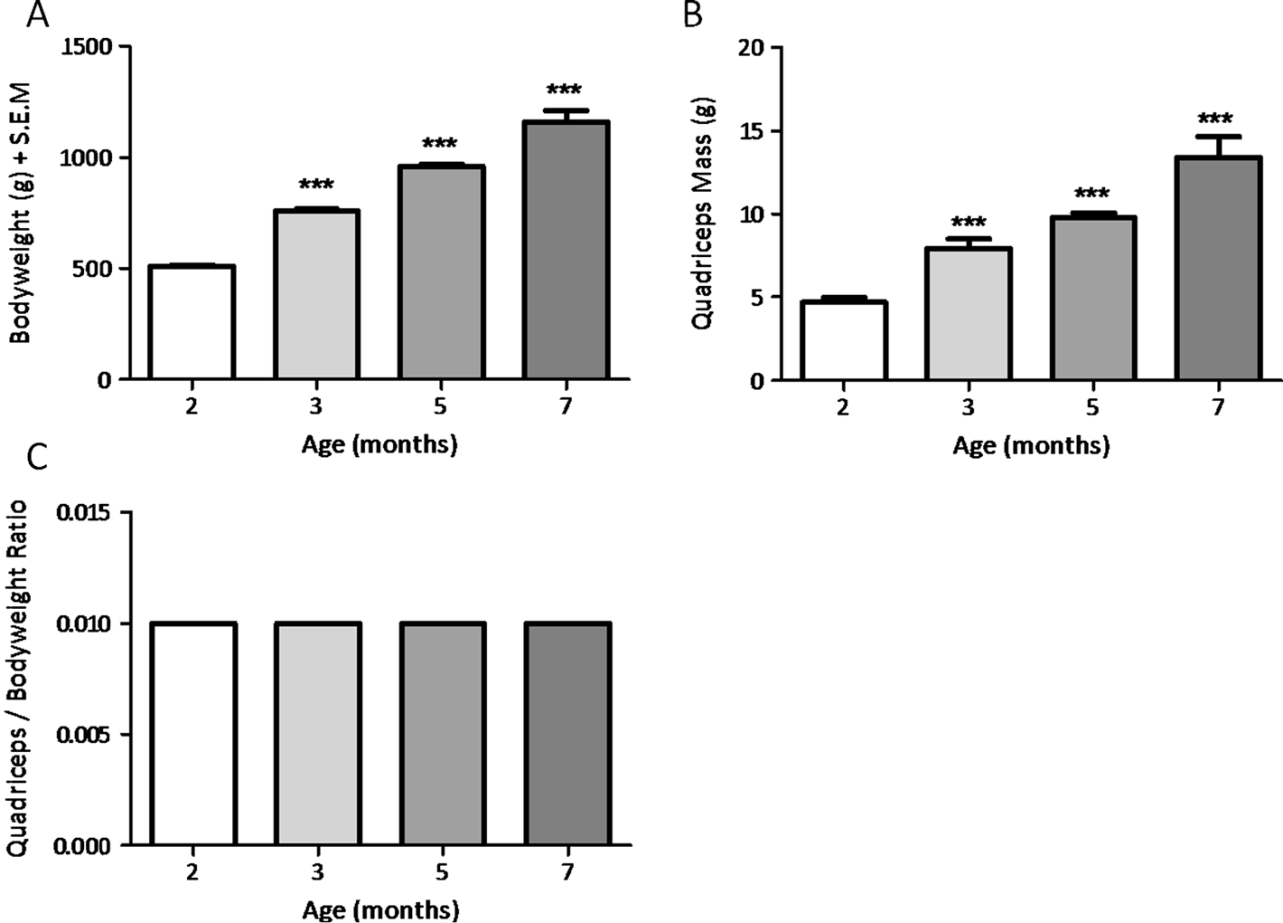
**The effect of advancing age on bodyweight (A), quadriceps mass (B) and quadriceps mass to bodyweight ratio (C).** Data are mean + SEM; n = 6; * denotes *P* <0.05, ** denotes *P* <0.01, *** denotes *P* <0.001.

All animals remained in good general health throughout the study and all 24 animals were included in the following analyses. All animals were group housed for the duration of the study and were active through their light phase. In line with the study animals being within their longitudinal growth phase, both body mass (g) and quadriceps mass (g) increased significantly with advancing age (*P* ≤0.001). Mean animal bodyweight progressed from 510.60 ±3.27 g at 2 months to 1160.78 ±48.72 g at 7 months of age (Figure 1a), whilst mean quadriceps mass increased from 4.68 ±0.28 at 2 months to 13.40 ±1.24 g at 7 months of age (Figure 1b). As an index of quadriceps hypertrophy or atrophy, a quadriceps to body mass ratio was determined [quadriceps mass (g) over body mass (g)]. Quadriceps mass relative to bodyweight remained constant at all ages (*P* = 1.000) (Figure 1c).

### Tibiofemoral pathology

**Figure 2:**
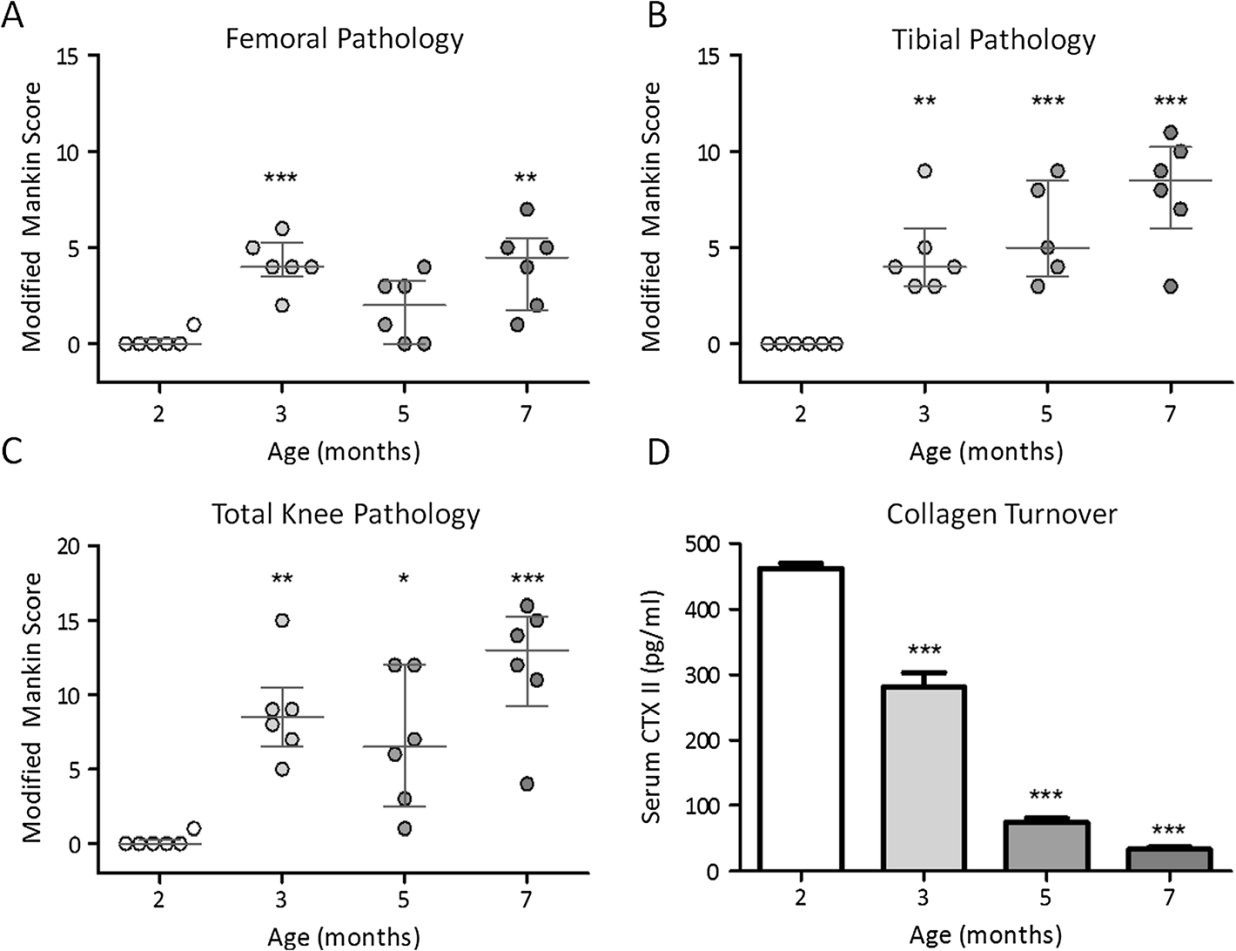
**Histological evidence of knee osteoarthritis on the femoral condyle (A), tibial condyle (B) and both condyles (C).** Data are modified Mankin scores; error bars denote median ± interquartile range. **(D)** Mean serum CTX II concentration (pg/mL); errors bars denote SEM; * denotes *P* <0.05, ** denotes *P* <0.01, *** denotes *P* <0.001. All groups were compared to the 2-month age group.

Histological examination of tibiofemoral joints was performed in accordance with previously validated methodology [35] and revealed an increase in joint pathology with advancing age. At 2 months of age, animals were generally free from knee OA with the exception of one animal that presented with mild proteoglycan loss in the superficial zone. Interestingly, the affected animal was the heaviest out of the 2-month cohort although it was still significantly lighter than any single animal assessed at 3 months of age. At 3 and 5 months of age, animals presented with proteoglycan loss extending as deep as the mid-zone and mild cartilage surface irregularities. At 7 months of age, proteoglycan loss and cartilage surface irregularities were more pronounced than at previous ages, although no animals exhibited osteophytosis at any of the joint margins studied (Figure 2a–c).

### Cartilage (collagen type II) degradation

Disruption of the structural integrity of articular cartilage is the major histological finding in OA and rheumatoid arthritis. Degradation products resulting from cartilage disruption include the terminal telopeptide of type II collagen (CTX II), which is released into the circulatory system [37]. Serum CTX II concentration decreased significantly with advancing age from 462.34 ±7.32 pg/mL at 2 months to 33.63 ±3.17 pg/mL at 7 months when the last study animals were assessed (*P* ≤0.001) (Figure 2d).

### Quadriceps femoris contractile parameters

The characteristics of skeletal muscles are a function of the contractile and metabolic properties of the muscle fibres from which they are composed. Contractile properties of the quadriceps skeletal muscle were assessed by the expression of myosin heavy chain (MHC) isoform mRNAs at each study time point as previously described [36,38]. Although many isoforms of MHC have been described, four are associated with adult skeletal muscle. One “slow-twitch” (Type I encoded by MyH7) muscle-associated MHC isoform and three “fast-twitch” (Types IIA, IIX and IIB encoded by MyH2, 1 and 4, respectively) muscle-associated isoforms. MHC mRNA expression has been previously shown to correlate well with both MHC protein abundance [39,40] and traditional histochemical measures of muscle fibre type [41].

**Figure 3:**
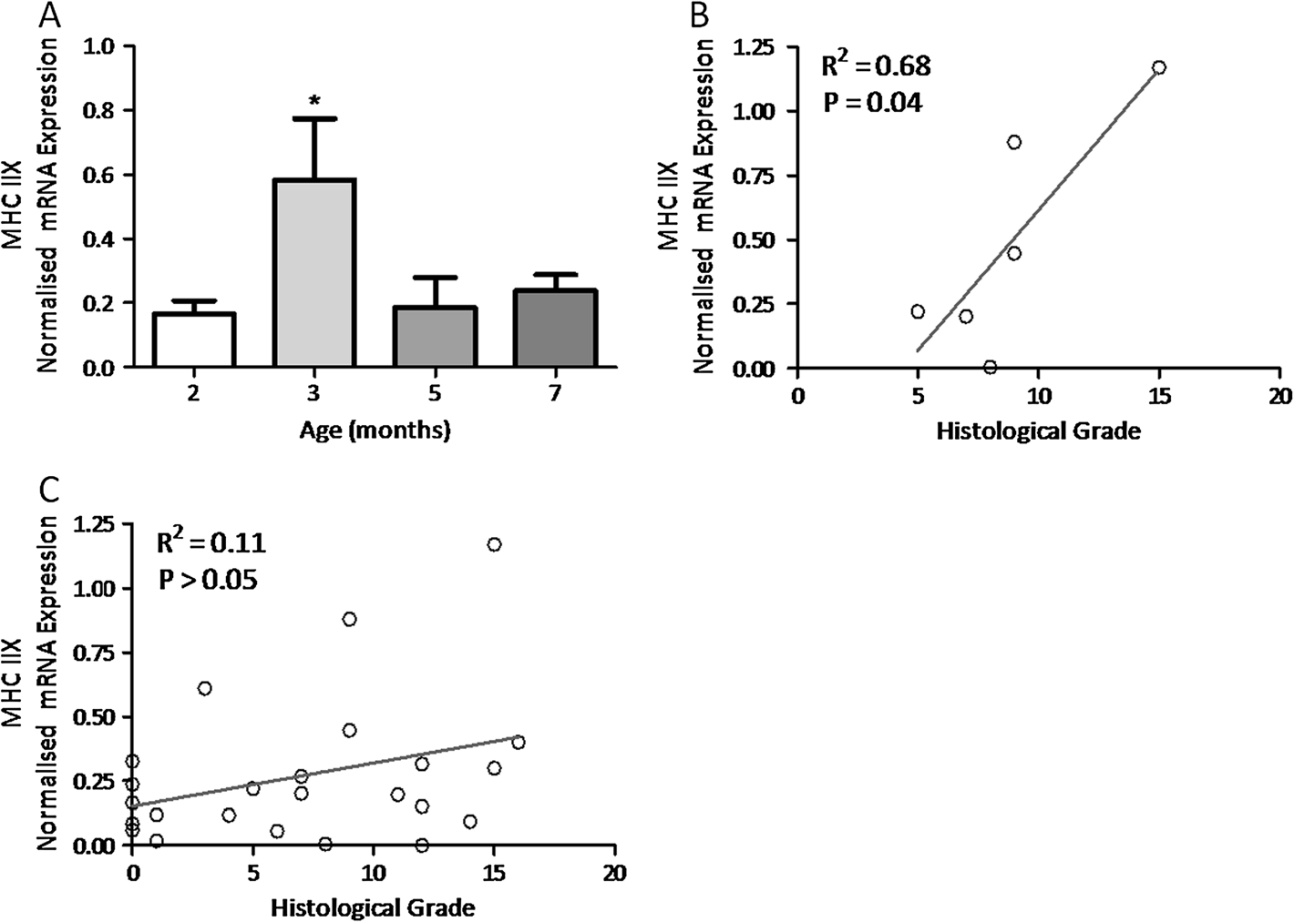
**Real-time PCR assessment of myosin heavy chain 1 (MHC IIX) isoform specific mRNA. (A)** MHC IIX mRNA expression in the quadriceps muscle of guinea pigs aged between 2 and 7 months. Data are mean expression units + SEM normalised to total first strand cDNA concentration; n = 6; * denotes *P* <0.05. **(B)** Linear regression analysis depicting the relationship between MHC IIX mRNA expression (y-axis) and total histological grade (x-axis) in guinea pigs at 3 months of age; n = 6. **(C)** Linear regression analysis depicting the relationship between MHC IIX mRNA expression (y-axis) and total histological grade (x-axis) in guinea pigs aged between 2 and 7 months; N = 24.

MHC I and IIA mRNA expression were unaltered as age advanced and OA developed (*P* = 0.117 and 0.627, respectively) (Table 2) suggesting that the associated slow-twitch postural-type muscle fibres were unaffected by OA development. Similarly, MHC IIB mRNA levels, associated with the fastest contracting muscle fibres remained unaltered with advancing age and developing pathology (*P* = 0.417) (Table 2). Interestingly, MHC IIX mRNA, associated with fast-twitch skeletal muscle fibres, was significantly elevated at 3 months of age coincident with the first evidence of OA (*P* = 0.038) (Figure 3). Furthermore, MHC IIX mRNA levels correlated positively with the total OA grade at this time point (R^2^ = 0.68, *P* <0.05), suggesting a trend between MHC IIX expression and disease severity. However, this relationship did not persist across all study time points (Figure 3).

**Table 2:** Real-time PCR assessment of myosin heavy chain (MHC) isoform specific mRNAs of MHC I, MHC IIa, MHC IIx and MHC IIb

**Parameter**	**2mo**	**3mo**	**5mo**	**7mo**	***P *** **value**
MHC I mRNA	0.195 ± 0.052	0.566 ± 0.362	0.034 ± 0.011	0.342 ± 0.300	0.117
MHC IIa mRNA	0.051 ± 0.008	0.077 ± 0.016	0.055 ± 0.023	0.051 ± 0.009	0.627
MHC IIx mRNA	0.165 ± 0.041	0.584 ± 0.191	0.184 ± 0.094	0.237 ± 0.050	0.038
MHC IIb mRNA	0.088 ± 0.015	0.084 ± 0.019	0.061 ± 0.024	0.051 ± 0.009	0.417

Data are mean expression units ± SEM normalised to total first strand cDNA concentration; n = 6.

**Figure 4:**
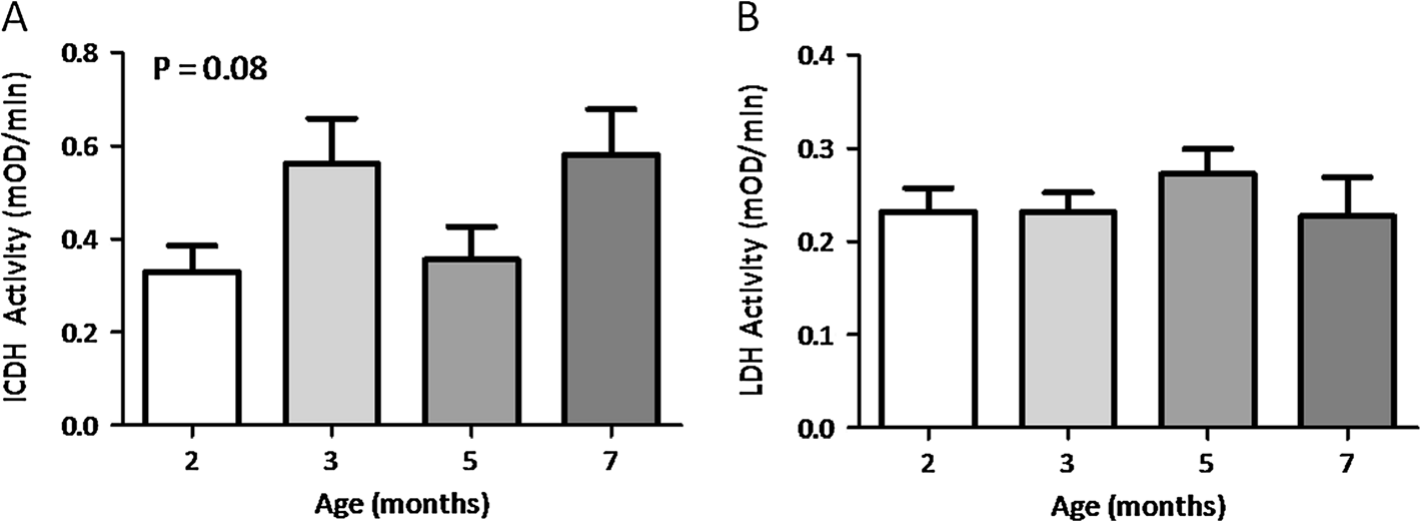
**ICDH (A) and LDH (B) enzyme activity in whole quadriceps homogenates.** Data are mean mOD/min normalised to total extractable protein; n = 6. Error bars denote SEM; *P* values refer to one-way analysis of variance.

**Figure 5:**
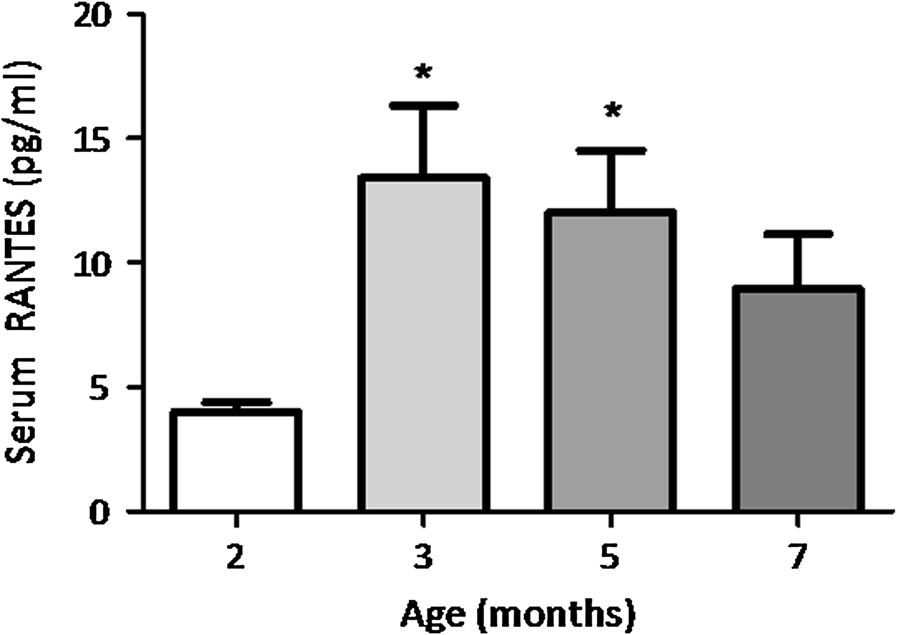
**Serum RANTES concentration (pg/mL) in guinea pigs between the ages of 2 and 7 months.** Data are mean serum concentration (pg/mL); n = 6. Error bars denote SEM; * denotes *P* <0.05.

An indication of the oxidative capacity of quadriceps skeletal muscle specimens associated with slow-twitch muscle fibres was determined by ICDH enzyme activity. Analysis of variance revealed a trend increase in activity (*P* = 0.08) with the most marked changes noted between the ages of 2 and 3 months, and 2 and 7 months (Figure 4). Interestingly, it was at these same time points that increased inter-animal variation was noted in MHC I mRNA expression (Table 2). An indication of glycolytic activity was determined in quadriceps specimens via the measurement of LDH enzyme activity. LDH activity was unaffected by age or the development of OA in this study (*P* = 0.867) (Figure 5).

### Serum RANTES expression

Elevated RANTES expression has been previously associated with active OA disease in human patients [42] and it was therefore of interest whether RANTES was elevated in our ageing model of OA. Circulating RANTES was significantly elevated at 3 months of age (approximately 3-fold the serum concentration seen at 2 months of age) coincident with the first histological evidence of OA *P* <0.05. Furthermore, serum RANTES was found to positively correlate with total osteoarthritic grade at this time point (R^2^ = 0.41, *P* = 0.16). Serum RANTES concentration did not correlate with any of the muscle parameters assessed in this study. The significant elevation in serum RANTES was maintained at 5 months of age (approximately 3-fold; *P* <0.05) and was still evident (although not significant) at 7 months of age (approximately 2-fold) when the final analyses were performed (Figure 5).

## Conclusions

This is the first study to investigate molecular factors associated with contractile and metabolic parameters of the quadriceps femoris skeletal muscle group during the age-associated primary onset of OA in the Dunkin Hartley guinea pig, and to associate these changes with the development and severity of knee OA. In order to fully characterise the development of ageing-associated disease, it is critical to assess the molecular and pathological changes that occur during disease initiation. This enables an understanding of the key molecular pathways that drive disease initiation in ageing models and permits the development of preventative therapeutics that aim to halt disease initiation rather than ameliorate symptoms or attempt to reverse established disease.

The histological features and timeframe of OA development in the Dunkin Hartley strain are generally well characterised [24-30]. Assessment of toluidine blue stained step coronal sections from guinea pigs in this study revealed that 2-month-old animals presented with histologically normal knee joints, whilst evidence of osteoarthritic-like lesions was present from as early as 3 months of age. Pathology was associated with reduced proteoglycan staining at the joint margin and changes to articular cartilage structure. In general, OA scores progressed concurrent with age through until 7 months of age when the final analyses were performed. The timing of OA initiation and development and total histological scores were concurrent with those of other published studies utilising the same strain and joint scoring system [35].

Coincident with the initial histological evidence of OA (at the age of 3 months), was a marked elevation in circulating RANTES (approximately 3-fold compared with 2-month-old OA-free animals) which was maintained until the final analyses were performed at 7 months of age. RANTES has been implicated in articular cartilage degradation by the enhancement of matrix metalloproteinase-3 production and suppression of proteoglycan in osteoarthritic chondrocytes [43]. Moreover, elevated serum RANTES concentrations have been specifically associated with active osteoarthritic disease when compared with healthy controls and people with established, non-active disease [43], suggesting that RANTES expression may play a role in the initial development of OA in this age-related disease model, similar to in humans.

Several publications report the potential utility of serum CTX II as a biomarker of OA [44,45] and it was measured in this study aiming to provide a more linear measure of OA severity than offered by traditional histopathological scoring techniques. Surprisingly, serum concentrations of CTX II significantly decreased as age advanced and the severity of osteoarthritic lesions detected increased although levels were concurrent with other published reports in the same strain [46,47]. The most likely explanation for this finding is that marked growth plate activity, associated with normal skeletal development in young animals, contributes significantly to the serum CTX II concentrations detected. Growth plate activity has been previously associated with markedly elevated serum CTX II concentrations [47-49] and levels have been shown to stabilise once animals reach skeletal maturity [48,49]. Growth plate contribution to serum CTX II load is reported to contribute until 6 months of age in rodents [48], 12 months of age in rabbits, and until 25 years of age in humans [50]. Taken together, these findings highlight the need to select skeletally-mature animals for use as spontaneous models of OA if measurements of cartilage turnover are required.

As anticipated, advancing guinea pig age was associated with both increased body mass and quadriceps skeletal muscle mass, which were significantly elevated between all of the time points studied. Although gross hypertrophic or atrophic effects to the quadriceps skeletal muscle were excluded on the basis of an unaltered quadriceps to body mass ratio, we sought to investigate whether any subtle molecular changes to this muscle group were associated with the primary onset of OA and its early progression in this model. Examination of factors indicative of contractile and metabolic properties of the quadriceps skeletal muscle revealed age-related effects on muscle fibre-type specific mRNAs. MHC IIX mRNA was elevated at 3 months of age (approximately 3.5-fold), coincident with the first histopathological sign of OA (*P* ≤0.05); furthermore, it was positively correlated with total pathology grade at this time. MHC IIX mRNA is associated with the expression of fast-twitch glycolytic muscle fibres and is the second fastest MHC isoform in many laboratory species including the mouse, rat [51] and guinea pig [36]. Conversely, MHC IIX is the fastest MHC isoform in the human [52], which generally lacks MHC IIB expressing muscle fibres. It is interesting to note that the elevated expression of MHC IIX mRNA, indicative of increased fast glycolytic muscle fibre expression, occurred at the time of OA initiation (at 3 months of age) before returning to basal levels thereafter. This finding could indicate altered skeletal muscle function around the time of OA initiation. In support of this, established OA has been previously associated with muscle fibre type changes in man [53,54] and in surgically-induced models [55]; however, this is the first report of such changes around the time of OA initiation in a guinea pig ageing model of OA.

There are a number of limitations in this study. The principal aim was to assess changes in molecular factors associated with skeletal muscle function in response to OA initiation in young animals, since understanding the key events during disease initiation in ageing models is important for the development of preventative therapeutics. However, since we studied young animals OA severity did not progress significantly during the time course studied and therefore further work is required using older animals over a protracted timeframe before any conclusions can be drawn on the potential role of sarcopenia in the progression of OA disease. As such, our preliminary findings of muscle changes in this ageing model of OA are predominantly applicable to early processes surrounding disease initiation.

Another potential limitation is the clinical relevance of the guinea pig model of OA. Although, we believe this model has many distinct advantages over surgically-induced rodent models, several caveats must be considered when translating any findings from preclinical animal models. Firstly, although there are many similarities to development of OA in humans, the development of OA in the Dunkin Hartley stain coincides with their longitudinal growth phase. This results in significant increases in body mass, which require careful control in time course studies. Furthermore, longitudinal growth is associated with active growth plate processes, negating the use of biomarkers of OA that rely on cartilage turnover. Due to the restricted availability of a suitable control strain which is sufficiently similar to the Dunkin Hartley strain but age without OA development, such studies are invariably cross-sectional, where findings are correlated to markers of disease severity.

Nevertheless, these preliminary findings suggest, for the first time, that initiation of OA in the guinea pig ageing model of OA occurs independently of gross changes to quadriceps muscle mass and that disease initiation is associated with changes in molecular factors indicative of altered muscle contractile properties. The suggestion that muscle quality rather than muscle mass is the primary determinant of disease is pertinent and warrants further investigation, including the assessment of physiological measures of muscle function to link our molecular observations to changes in skeletal muscle functional output. Understanding the key molecular pathways that drive disease initiation in ageing models is essential for the development of novel preventative therapeutics. However, such observations should be conducted over a longer period if a relationship between skeletal muscle dysfunction and sarcopenia with OA disease progression is to be established.

## Methods

### Animals, housing and study design

Male Dunkin Hartley guinea pigs (N = 24) were sourced from Charles Rivers, UK, at 6 weeks of age. Animals were group-housed in large pens (4 m x 8 m) with free access to standard guinea pig chow (Purina, UK) and water. At 2, 3, 5 and 7 months of age, six animals were selected based upon their proximity to the median weight of the cohort and euthanized as described below. All animal procedures underwent ethical approval by the University of Nottingham and were conducted in full compliance with the Animals (Scientific Procedures) Act, 1986.

### Termination and histopathology

Animals were euthanized by intra-peritoneal injection of pentobarbital sodium and death was confirmed by cervical dislocation. Knee joints were obtained for histopathological analysis by making a full thickness cut 2 cm above and below the patella. The joints were formalin fixed and decalcified in 10% formic acid prior to processing by routine vacuum assisted wax infiltration. Toluidine blue stained step coronal sections were prepared at 300 μm intervals and evaluated using a histological scoring system optimised and validated for guinea pig specimens [35]. Pathological features at each condyle were combined to calculate a femoral, tibial and combined OA score. The observer was blinded to both the animal number and age in all cases.

### Biospecimens

Whole bilateral quadriceps muscle samples, inclusive of the rectus femoris, were dissected, weighed and immediately snap frozen in isopentane cooled with liquid nitrogen. Care was taken to avoid inclusion of any adipose tissue or additional muscle, most importantly the tensor fasciae latae and sartorius, which are located within the dissected area. Whole blood was drawn via cardiac puncture into clot-activator tubes (Sarstedt) and serum was obtained by centrifugation. All serum was kept at −80°C prior to analysis.

### Extraction of total RNA

Total RNA was extracted from 100 mg of sample using TRIzol regent (Invitrogen) according to standard procedure. Contaminating genomic DNA was removed by RQ RNase-Free DNase I digestion (Promega) as specified by the manufacturer’s standard instructions. The resulting total RNA was re-suspended in molecular biology grade water (Promega). All RNA was stored at −80°C prior to use.

### Reverse transcription

First strand complementary DNA (cDNA) was reverse transcribed from 1 μg total RNA using random hexamers and Moloney murine leukemia virus reverse transcriptase (MMLV) in a final volume of 25-μL as described by the manufacturer (Promega).

### Primer design

Previously published oligonucleotide primers [36] were sourced from MWG Eurofins Operon (Table 1).

### Quantitative PCR

Quantitative PCR reactions were performed in triplicate on 5 μL cDNA in SYBR 1 Master mix (Roche), 0.25 mM forward and reverse primers in a final volume of 15 μL. Cycling parameters were 95°C for 5 minutes prior to 35 cycles of 10 seconds at 95°C, 10 seconds at 55°C and 30 seconds at 72°C. Single signal acquisition was set to read at 72°C. All reactions were run on a 384-well microplate on a LightCycler LC480 (Roche) configured for SYBR green determination as specified by the manufacturers. Melt curve analysis was performed at the end of each completed analysis run to ensure only the specific product was amplified. All quantitative PCR data was normalised to the total first strand cDNA concentration following reverse transcription using OliGreen (Invitrogen).

### Serum CTX II assessment

Serum CTX II concentration was determined by a validated enzyme linked immunosorbent assay incorporating a monoclonal antibody specific for the neo-epitope formed when collagen type II is degraded to form CTX II (Serum Cartilaps, IDS, USA). Samples were processed according to the manufacturer’s instructions using 25 μL of guinea pig serum against standards produced from rat CTX II of known concentrations (0–247.6 pg/mL). All samples were analysed in duplicate and a coefficient of variation <5% was deemed acceptable.

### Skeletal muscle metabolic potential

Isocitrate dehydrogenase (ICDH) and lactate dehydrogenase (LDH) enzyme activities were measured as an index of oxidative (aerobic) metabolism and glycolytic (anaerobic) metabolism, respectively. Both enzyme activities were measured in accordance with the original method of Brandstetter, 1998 [56].

### Serum regulated upon activation, normal T-cell expressed and secreted (RANTES) assessment

Serum RANTES expression was determined by fluorescent enzyme-linked immunosorbent assay (ELISA) (BioRad). Serum samples from all guinea pigs were analysed as recommended by the manufacturer against a range of rat cytokine standards (0–3,200 pg/mL) and a sample dilution of 1:3, utilising a total of 30 μL of sera. All samples were analysed (Bio-Plex 200) in triplicate, with a coefficient of variation <5% deemed as acceptable.

### Statistical analysis

All data are reported as mean ± standard error of the mean (SEM) unless otherwise specified. Comparisons between multiple groups were performed by analysis of variance (ANOVA) using GraphPad software V5.0 (Prism) with Dunnett’s post hoc test (comparing all experimental groups to the 2 month group) performed where *P* <0.05.

## Abbreviations

CTX II: Telopeptide of type II collagen; ICDH: Isocitrate dehydrogenase; LDH: Lactate dehydrogenase; MHC: Myosin Heavy Chain; OA: Osteoarthritis.

## Competing interests

This work was funded by the Arthritis Research Campaign for PhD funding (Grant RB3563) and AstraZeneca, Macclesfield. The authors declare that they have no competing interests.

## Authors’ contributions

DPT performed the experiments and prepared the manuscript. DPT, RGB, RAM, TP and SWJ were involved in the interpretation of data and strategic planning. All authors read and approved the final manuscript.
